# Allelic Imbalance in the miR-31 Host Gene Locus in Lung Cancer - Its Potential Role in Carcinogenesis

**DOI:** 10.1371/journal.pone.0100581

**Published:** 2014-06-30

**Authors:** Koji Okudela, Yoko Tateishi, Shigeaki Umeda, Hideaki Mitsui, Takeshisa Suzuki, Yuichi Saito, Tetsukan Woo, Michihiko Tajiri, Munetaka Masuda, Yohei Miyagi, Kenichi Ohashi

**Affiliations:** 1 Department of Pathology, Yokohama City University Graduate School of Medicine, Yokohama, Japan; 2 Department of Surgery, Yokohama City University Graduate School of Medicine, Yokohama, Japan; 3 Division of General Thoracic Surgery, Kanagawa Cardiovascular and Respiratory Disease Center Hospital, Yokohama, Japan; 4 Clinical Research Institute, Kanagawa Prefectural Cancer Center Hospital, Yokohama, Japan; Institute of Hepatology - Birkbeck, University of London, United Kingdom

## Abstract

Small non-protein coding RNA, microRNA (miR), which regulate messenger RNA levels, have recently been identified, and may play important roles in the pathogenesis of various diseases. The present study focused on miR-31 and investigated its potential involvement in lung carcinogenesis. The expression of miR-31 was altered in lung cancer cells through either the amplification or loss of the host gene locus. The strong expression of miR-31 in large cell carcinomas was attributed to the gene amplification. Meanwhile, the loss of miR-31 expression was more frequently observed in aggressive adenocarcinomas. Thus, miR-31 may play a pleiotropic role in the development of lung cancers among different histological types. To the best of our knowledge, this is the first study to show the potential causative mechanism of the altered expression of miR-31 and suggest its potentially diverse significance in the different histological types of lung cancers.

## Introduction

Lung cancer is one of the most common causes of cancer-related death in the developed world [1], [2]. Even if the primary tumor is successfully resected, a recurrence is observed in a large percentage of patients [1], [2]. Although some lung tumors are sensitive to conventional chemotherapeutic agents or certain molecular targeting agents, many are not [3], [4]. Thus, further understanding of the molecular mechanism underlying lung carcinogenesis is important for the development of novel therapeutic strategies.

Small non-protein coding RNA, microRNA, which regulate the messenger RNA levels, have recently been identified, and has been shown to play important roles in the pathogenesis of various diseases. Various microRNA (miR), including let-7, miR-21, miR-30d, miR-31, miR-155, and miR-205, have been suggested to be involved in the carcinogenesis of different types of malignancies [5]. Among them, miR-31 was reported to be more strongly expressed in tumor tissue than in non-tumorous tissue, and was suggested to have an oncogenic role in lung carcinogenesis [6]–[12]. Moreover, a recent study demonstrated the prognostic value of miR-31, because the higher expression of miR-31 was associated with a poorer outcome in patients with lung cancer [13]. Meanwhile, a difference in miR-31 expression among histological types of lung cancers and the potential mechanism of an altered expression of miR-31, have not been elucidated.

The present study analyzed the expression of miR-31 and the status of its host gene locus in the different histological types of lung cancers.

## Materials and Methods

### Cell lines and culture

An immortalized human airway epithelial cell line (16HBE14o, Simian virus 40 (SV40)-transformed human bronchial epithelial cells) described by Cozens AL et al. (1994) [14] was kindly provided by Grunert DC (California Pacific Medical Center Research Institute). A sub-clone of 16HBE14o cells, described as NHBE-T in this study, was used. Immortalized airway epithelial cell lines (HPL1D and HPL1A, SV40-transformed human small airway epithelial cells) were established by Masuda A et al. (1997) [15]. Human lung cancer cell lines (A549, H322M, H358, H522, H820, H2087, H23, EKVX, H226, H827, H1819, H441, H4006, HOP62, H1299, and H460) and a human embryonic kidney cell line (HEK293T) were purchased from the American Type Culture Collection (ATCC, Manassas, VA). The human lung cancer cell line LC2AD, Lu130, Lu135, Lu139, and Lu140, was purchased from the Riken Cell Bank (Tsukuba, Japan). The human lung cancer cell lines, PC9 and HARA, were from Immuno-Biological Laboratories Co. (Gunma, Japan). The human lung cancer cell lines, TKB1, TKB2, TKB4, TKB5, TKB6, TKB7, TKB8, TKB9, TKB12, TKB14, TKB15, TBK17, and TKB20, were obtained from Dr. Hiroshi Kamma via Dr. Takuya Yazawa (Kyorin University School of Medicine) [16]. Primary small airway epithelial cells (SAEC) and normal human bronchial epithelial cells (NHBE) were purchased from SANKO Kagaku (Tokyo, Japan).

### Primary lung Cancer

A total of 129 primary lung tumors (71 adenocarcinomas, 38 squamous cell carcinomas, 18 large cell carcinomas, 2 small cell carcinomas) were removed by radical surgical resection at Kanagawa Cardiovascular and Respiratory Center Hospital (Yokohama, Japan). This study was performed in compliance with the Helsinki Declaration [17], and was approved by the Ethics Committees of Yokohama City University and Kanagawa Prefectural Cardiovascular and Respiratory Center Hospital [18]. Written informed consent was obtained from all subjects providing materials.

### RNA extraction

Cell lines were washed with cold phosphate buffer saline and then snap frozen. Formalin-fixed and paraffin-embedded tissue sections were examined microscopically. Tumorous and non-tumorous parts were dissected with a razor blade. Total RNA was extracted using the miRNeasy mini kit (Qiagen, Valencia, CA).

### Quantitative RT-PCR

To detect miRNA, first-strand cDNA was synthesized from total RNA using the Mir-X miRNA First-Strand Synthesis Kit according to the protocols of the manufacturer (Takara, Kyoto, Japan). The cDNA generated was used as a template in real-time PCR with SYBR Premix EXTaq (Takara) and run on a Thermal Cycler DICE real-time PCR system (Takara). The forward primer used for the detection for miR-31 was 5′- AGGCAAGATGCTGGCATAGCT (mature miR-31, MIMAT0000089). The reverse primer was the universal mRQ 3′ primer (Takara). The primer set used to detect U6 snRNA was purchased from Takara Bio Inc. MiR-31 level was normalized to U6 snRNA levels. To detect mRNA, first-strand cDNA was synthesized from total RNA using the SuperScript III First-Strand Synthesis System (Invitrogen). The cDNA generated was used as a template in real-time PCR with TaqMan Gene Expression Assay system (Applied Biosystems, Foster City, CA) and run on a Thermal Cycler DICE real-time PCR system (Takara). The TaqMan probes and primer set used to detect GAPDH [NM_002046.5], ITGA5 [NM_002205.2], MMP16 [NM_005941.4], and RHOA [NM_001664.2], was customized and purchased Applied Biosystems. ITGA5, MMP16, and RHOA levels were normalized to GAPDH levels.

### PCR analysis of the miR-31 locus

Genomic DNA was extracted from the cancer cell lines using the DNeasy kit (Qiagen). The status (deletion or retention) of the miR-31 hot gene locus was analyzed by genomic DNA PCR using the primer set of F 5′- TAACTACATCTTCAAAAGCGGAC and R 5′- TACATAGCAGGACAGGAAGTAA. The status of the CDKN2A gene locus (D9S974) and another distant locus of the short arm of chromosome 9 (D9S304) were also analyzed using the primer sets of F 5′- GAGCCTGGTCTGGATCATAA and R 5′- AAGCTTACAGAACCAGACAG, and F 5′- GTGCACCTCTACACCCAGAC and R 5′- TGTGCCCACACACATCTATC, respectively.

### *In situ* hybridization for miRNA

*In situ* hybridization was performed according to a method described previously [19]. Briefly, the locked nucleic acid (LNA)-modified detection probes for miR-31 and U6 snRNA, and scrambled negative control probe (Exiqon, Vedbaek, Denmark) were labeled with digoxigenin (DIG) using the DIG Oligonucleotide Tailing kit (Roche, Basel, Switzerland) according to the manufacturer's instructions. Formalin-fixed and paraffin-embedded tissue sections were acetylated and hybridized with DIG-labeled detection probes, and were then probed with alkaline phosphatase-conjugated anti-DIG Fab fragments (Roche). The hybridization signal was visualized using a color developer solution (Roche).

### Fluorescent *in situ* hybridization (FISH) for the gene locus

Formaldehyde-fixed and paraffin-embedded tissue sections were used for the analysis. Sections were boiled in citrated buffer (0.01 M, pH 6.0) to release closed chromosomal structures [20]. These sections were hybridized with a Cy3-labeled probe covering the miR-31 locus (BAC clone: RP11-354P17) and a FITC-labeled probe for the centromere locus on chromosome 9 (Vysis INC., Downer Groves, IL). The signal from the miR-31 locus and centromere 9 was counted for more than 50 cells in every tumor, and the copy number of the miR-31 locus relative to that of centromere 9 was calculated.

### Treatment with 5-azacytidine and trichostatin A

Cells were treated either with 10 µM of 5-azacytidine (Sigma, St. Louis, MO) for 72 hours by exchanging the medium every day or with 300 ng/ml of trichostatin A (Wako, Osaka, Japan) for 24 hours. Cells were also treated with 5-azacytidine for 48 hours and then with a combination of 5-azacytidine and trichostatin A for an additional 24 hours.

### Methylation-specific PCR

Genomic DNA was subjected to a bisulfate conversion treatment using the MethylEasy DNA bisulfate modification kit (Human Genetic Signatures, Macquarie Park, Australia). Methylation-specific PCR targeting the two CpG sites in the promoter locus of the miR-31 host gene (LOC554202), which was previously demonstrated [21], was performed according to the method described elsewhere [21].

### Bisulfate DNA Sequencing

A GpC site in the promoter locus of the miR31 host gene (LOC554202), which was previously demonstrated [21], was PCR-amplified using bisulfate-treated genomic DNA as a template, according to the method described elsewhere [21]. The PCR product was sub-cloned into the pT7 blue plasmid vector (Novagen, Darmstadt, Germany), and was then subjected to a cycle dye-terminator reaction with the universal T7 promoter primer using the Big Dye Terminator Version 3.1 kit (Applied Biosystems, Foster City, CA).

### Statistical Analysis

Differences in the means of the relative copy number of the miR-31 locus among groups classified according to various pathological parameters were analyzed with one-way ANOVAs. Differences in the means of miR-31 levels in the inhibitory experiment were analyzed with an unpaired Student's t test. Statistical analyses were performed using SPSS software (SPSS for Windows Version 10.0, SPSS, Chicago, IL, USA).

### Others

The experimental procedures used to perform immunohistochemistry for Ki-67 and analyze oncogenic mutations in the KRAS and EGFR genes were described elsewhere [22].

## Results

### MiR-31 and the expression of its known targets in lung cancer cell lines

**Figure 1:**
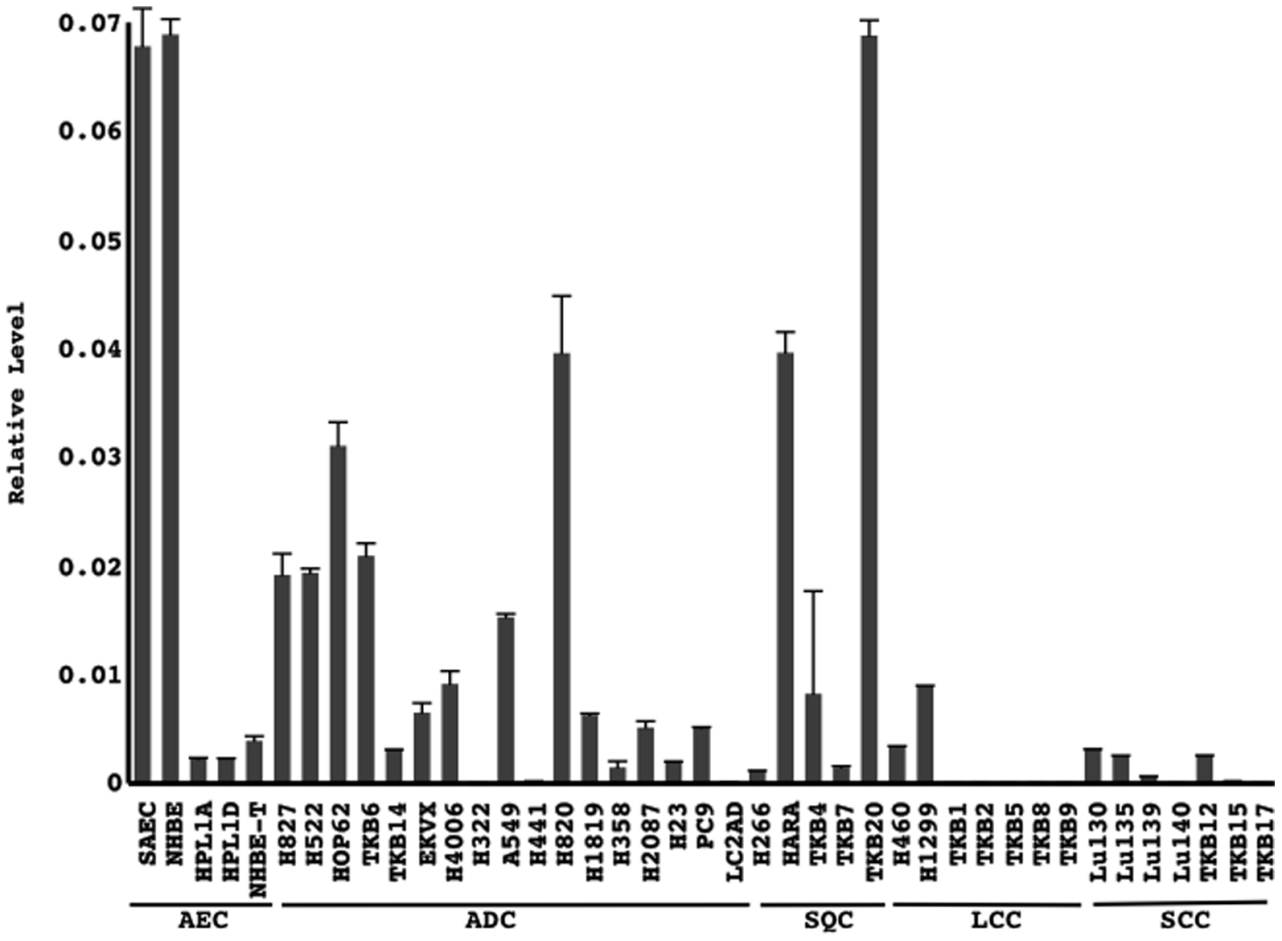
The copy numbers of miR-31 and U6 snRNA were measured using quantitative RT-PCR. MiR-31 levels were normalized to U6 snRNA levels. Technical replicates were performed in triplicate. Means and standard deviations (error bars) are shown. AEC, airway epithelial cells (non-cancerous cells); ADC, adenocarcinoma; SQC, squamous cell carcinoma; LCC, large cell carcinoma cell; SCC, small cell carcinoma.

MiR-31 expression appeared to be strong in some cancer cell lines, but was completely absent in other some cell lines (Fig. 1). The expression of let-7i was also examined. Let-7i was expressed in all the cell lines and its levels differed from those of miR-31 (Figure S1). It served as a control to assess the quality of the materials and ensured that marked alterations occurred in the expression of miR-31. Moreover, a preliminary experiment demonstrated that the restoration of miR-31 markedly suppressed the growth of a cancer cell line (LC2AD) that almost lost its ability to express miR-31 (Figure S2). These results prompted us to further investigate the potential significance of the altered expression of miR-31 in lung carcinogenesis.

### Status of the miR-31 gene locus in lung cancer cell lines

**Figure 2:**
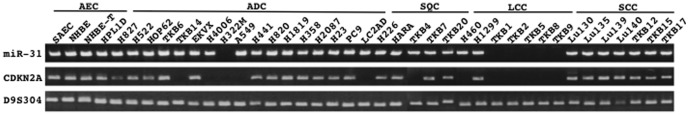
Lung cancer cell lines were examined for the status of the miR-31 gene locus, and also the statuses of the CDKN2A locus and another site of the short arm of chromosome 9 (D9S304). The results from PCR analysis are shown. AEC, airway epithelial cells (non-cancerous cells); ADC, adenocarcinoma; SQC, squamous cell carcinoma; LCC, large cell carcinoma cell; SCC, small cell carcinoma.

The statuses of the miR-31 gene locus, CDKN2A locus, and the adjacent locus (D9S304), were investigated using PCR analysis. Among the forty-one cell lines examined, six cell lines (14.6%, 6/41 cell lines; H322, TKB1, TKB2, TKB5, TKB8, and TKB9) lost the miR-31 host gene locus (Fig. 2, Table 1), while twelve lines (29.3%, 12/41 cell lines; TKB14, H4006, A549, LC2AD, TKB4, H460, H322, TKB1, TKB2, TKB5, TKB8, TKB9) lost the CDKN2A locus (Fig. 2, Table 1). All six lines that lost the miR-31 locus also lost the CDKN2A locus (Fig. 2, Table 1). The adjacent locus (D9S304) served as a positive control for PCR analysis (Fig. 2).

**Table 1:** miR-31 expression and statuses of the miR-31 and CDK2NA gene loci in non-cancerous airway epithelial cell and lung cancer cell lines.

Cell	Histology	miR-31 exp	miR-31 lcs	CDK2NA lcs
SAEC	AEC	+	+	+
NHBE	AEC	+	+	+
NHBE-T	AEC	+	+	+
HPL1A	AEC	+	+	+
HPL1D	AEC	+	+	+
H827	ADC	+	+	+
H522	ADC	+	+	+
HOP62	ADC	+	+	+
TKB6	ADC	+	+	+
TKB14	ADC	+	+	-
EKVX	ADC	+	+	+
H4006	ADC	+	+	-
H322M	ADC	-	-	-
A549	ADC	+	+	-
H441	ADC	-	+	+
H820	ADC	+	+	+
H1819	ADC	+	+	+
H358	ADC	+	+	+
H2087	ADC	+	+	+
H23	ADC	+	+	+
PC9	ADC	+	+	+
LC2AD	ADC	-	+	-
H226	SQC	+	+	+
HARA	SQC	+	+	+
TKB4	SQC	+	+	-
TKB7	SQC	+	+	+
TKB20	SQC	+	+	+
H460	LCC	+	+	-
TKB5	LCC	-	-	-
TKB2	LCC	-	-	-
TKB1	LCC	-	-	-
H1299	LCC	+	+	+
TKB8	LCC	-	-	-
TKB9	LCC	-	-	-
Lu130	SCC	+	+	+
Lu135	SCC	+	+	+
Lu139	SCC	+	+	+
Lu140	SCC	-	+	+
TKB12	SCC	+	+	+
TKB15	SCC	-	+	+
TKB17	SCC	-	+	+

exp, expression; lcs, locus; AEC, non-cancerous airway epithelia; ADC, adenocarcinoma; SQC, squamous cell carcinoma; LCC; large cell carcinoma; SCC, small cell carcinoma; +, detectable expression or retained gene locus; -, loss of expression or gene locus; Cell lines in which expression of miR-31was absent are underlined.

### Epigenetic modification of the miR-31 promoter and its expression

**Figure 3:**
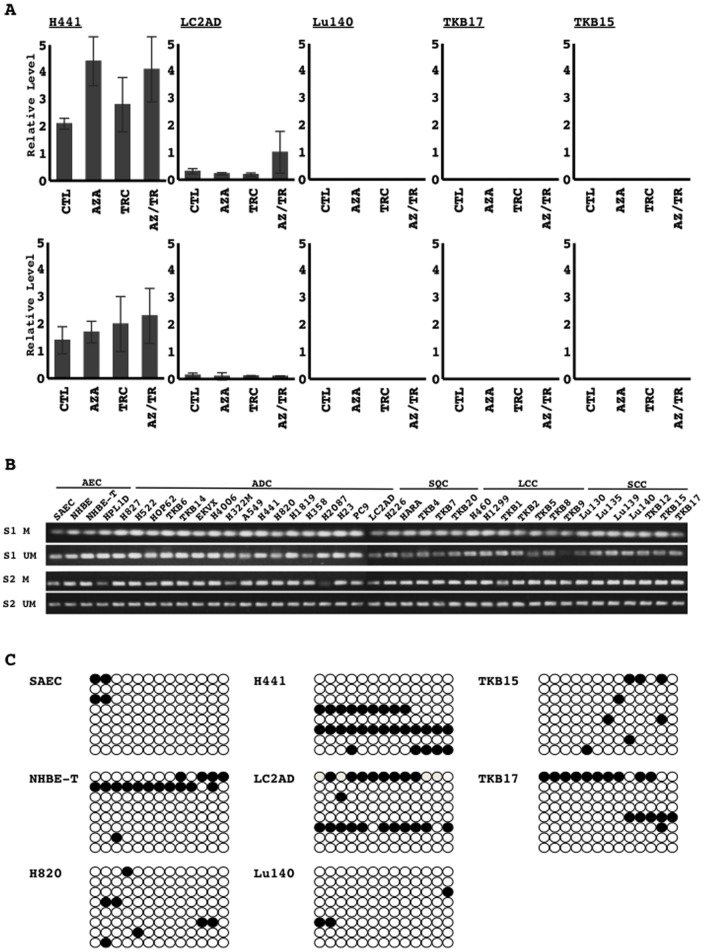
The possible involvement of epigenetic modifications in the downregulation of miR-31 in lung cancer cell lines was investigated. Cells treated with vehicle control (CTL), 5-azacytidine (AZA), trichostatin A (TRC), or a combination of AZA and TSA (AZ/TR) were examined for the restoration of miR-31 expression using quantitative RT-PCR. The relative level of miR-31 normalized to those of U6 snRNA was calculated. Two independent experiments were performed. Technical replicates of PCR analysis were performed in triplicate. Means and standard deviations (error bars) are shown (A). The two sites (S1 and S2) from the putative promoter of miR-31 were PCR-amplified with primers specific for either methylated (M) or unmethylated (UM) DNA using sodium bisulfate-modified genomic DNA as a template, according to the method described in a previous study [21]. Representative results are presented (B). The region of the miR-31 promoter containing CpG sites was PCR-amplified using sodium bisulfate-modified genomic DNA as a template, according to the method described in a previous study [21]. Eight subclones of PCR products were analyzed for their methylation status using DNA sequencing. Open (○) and filled (•) circles indicated unmethylated and methylated cytosine at the indicated CpG sites, respectively (C). AEC, airway epithelial cells (non-cancerous cells); ADC, adenocarcinoma; SQC, squamous cell carcinoma; LCC, large cell carcinoma; SCC, small cell carcinoma.

MiR-31 expression was absent in all the six cell lines losing the miR-31 host gene locus (Fig. 2, Table 1). Meanwhile, the five cell lines (H441, LC2AD, Lu140, TKB15, and TKB17) that retained the miR-31 gene locus (Fig. 2, Table 1) also lost miR-31 expression or markedly reduced its level (Fig. 2, Table 1), which indicated the potential involvement of epigenetic modification in its downregulation. Thus, the cell lines that lost miR-31 expression, but retained its gene locus were examined to investigate the effects of inhibitors for DNA methyltransferase and histone deacetylase on the expression of miR-31. Either and/or the combination of both inhibitors did not reproducibly restore miR-31 expression in these cells (Fig. 3A). Methylation-specific PCR analysis on two sites in the promoter region of the miR-31 host gene revealed that the two sites were methylated in all cell lines examined (Fig. 3B). Bisulfate DNA sequencing analysis on the CpG site of the promoter region also revealed that it was occasionally methylated in all cell lines examined (SAEC, NHBE-T, H820, H441, LC2AD, Lu140, TKB15, and TKB17) (Fig. 3C). The levels of methylation appeared to be not significantly different between cells retaining the expression of miR-31 (SAEC, NHBE-T, and H820) and those losing it (H441, LC2AD, Lu140, TKB15, and TKB17) (Fig. 3C). The methylation status of the putative promoter regions of the miR-31 host gene was not associated with the recovery status of miR-31 levels following the treatments with the inhibitors (Fig. 3A and 3C). Thus, DNA methylation in the putative region examined here could not be attributed to the downregulation of miR-31.

### Expression of miR-31 in primary lung cancers

**Figure 4:**
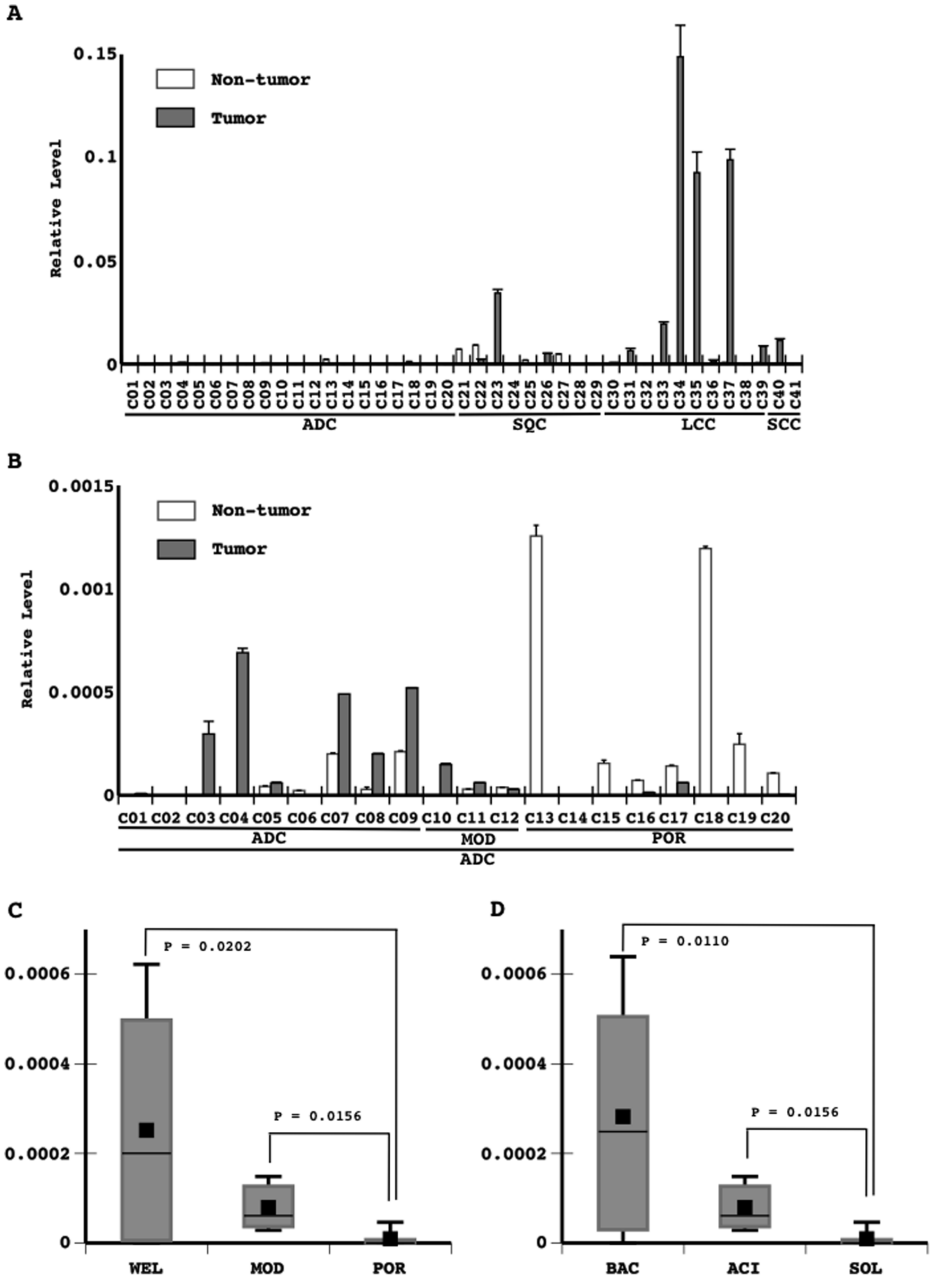
The expression of miR-31 was examined in primary lung tumors (20 adenocarcinomas (ADC), 9 squamous cell carcinoma (SQC), and 10 large cell carcinomas (LCC), which comprised some of the 127 non-small cell lung cancers listed in **Table 2, and 2** small cell carcinomas (SCC)). MiR-31 levels were evaluated in primary lung tumors and the corresponding lung non-tumorous tissue. The copy numbers of miR-31 and U6 snRNA were measured using quantitative RT-PCR. Technical replicates were performed in triplicate. The means and standard deviations (error bars) of miR-31 levels normalized to those of U6 snRNA are shown as the results obtained from all tumors (A) and those of ADC (B). Significant differences in the mean miR-31 levels in adenocarcinomas among the different histological grades (9 well differentiated carcinomas (WEL); 3 moderately differentiated carcinomas (MOD); 8 poorly differentiated carcinomas (POR)) and subtypes (8 bronchioloalveolar carcinoma (BAC); 3 acinar carcinoma (ACN); 8 solid carcinoma (SOL)(one papillary carcinoma ( =  well differentiate carcinoma) was excluded from a statistical analysis) were analyzed with a one-way ANOVA. The means and standard deviations (error bars) of the histological grades (C) and subtypes (D) are presented.

Quantitative RT-PCR analysis revealed that some tumorous and non-tumorous tissues expressed miR-31 at detectable levels (Fig. 4A). These levels varied, but were markedly high in some of the tumors examined (Fig. 4A). These appeared to be slightly higher in large cell carcinomas and slightly lower in adenocarcinomas (Fig. 4A). Among the adenocarcinomas examined, the expression of miR-31 was lower in poorly differentiated carcinoma (Fig. 4B and 4C) as well as in the solid subtype (Fig. 4D). Severe tissue damage accompanied by regenerative reactions was observed in non-tumorous tissues expressing miR-31 (not shown).

**Figure 5:**
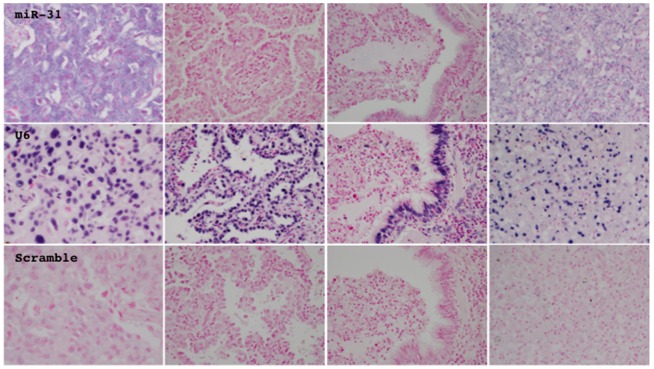
The expression and localization of miR-31 (top panels) and U6 snRNA (middle panels) were investigated by *in situ* hybridization analysis. A probe consisting of the scrambled random sequence served as a negative control (bottom panels). Representative photographs from tumorous (left two panels) and non-tumorous tissue (right two panels), which expressed miR-31 (center two panels) or did not (lateral two panels), are presented.

*In situ* hybridization analysis for miR-31 on tissue sections confirmed that miR-31 was expressed in neoplastic cells and also revealed that it even varied among neoplastic cells even in the same tumor (Fig. 5). Regenerative epithelial cells, interstitial cells, and inflammatory cells also expressed miR-31 at various levels (Fig. 5).

### Status of the miR-31 host gene locus in primary lung cancers

**Figure 6:**
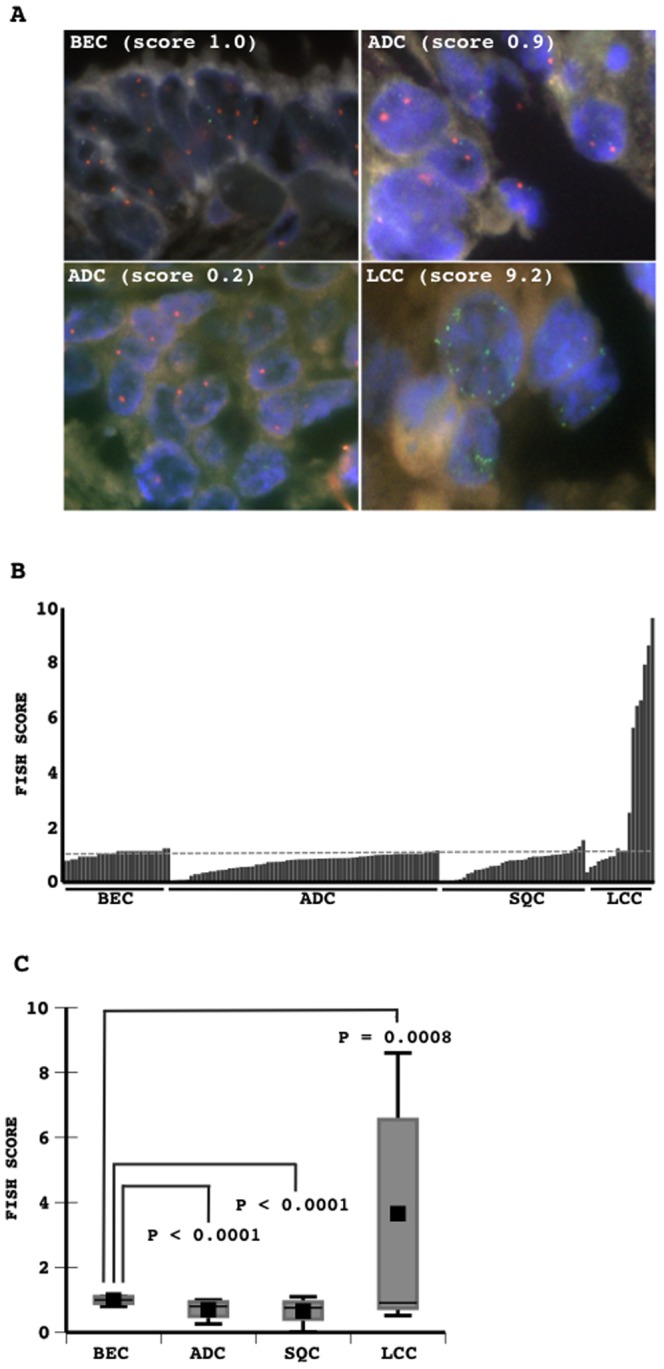
Primary lung tumors were examined for the status of the miR-31 host gene locus using fluorescent *in situ* hybridization. Representative results from tumorous and non-tumorous tissues are presented, and green and red signals indicate the miR-31 locus and centromere locus of chromosome 9, respectively (A). The signal count from the miR-31 locus normalized to that from the centromere locus of chromosome 9 was determined as the FISH score. The FISH scores measured are presented (B). The mean scores in non-tumorous bronchial epithelia and in the different histological types of lung cancers are presented (C). Differences in the mean score were analyzed with a one-way ANOVAs test. P values are indicated. BEC, bronchial epithelial cells (non-cancerous cells); ADC, adenocarcinoma; SQC, squamous cell carcinoma; LCC, large cell carcinoma.

FISH analysis revealed that the miR-31 host gene locus was lost in the neoplastic cells of some tumors, as the signal count from the miR-31 locus was lower than that from the centromere of chromosome 9 (Fig. 6A). The gene dosage of the miR-31 locus estimated by the ratio of miR-31/centromeres in adenocarcinomas and squamous cell carcinomas was slightly lower than that in non-cancerous bronchial epithelia (Fig. 6B). However, in some tumors of large cell carcinomas the miR-31 locus was amplified (Fig. 6C). The gene dosage was significantly higher in large cell carcinomas than in non-cancerous epithelia and other histological types (Fig. 6C, Table 2). All tumors with an amplification of the gene locus, that were availably examined, expressed a high level of the miR-31 transcript. In contrast, among adenocarcinomas, the gene dosage further tended to be lower in more aggressive tumors (poorly differentiated tumors or solid subtype tumors) (Table 2).

**Table 2:** Correlation between the status of the miR-31 host gene locus and pathologic subjects.

Subjects	FISH score
*Histology (127)	
ADC (71)	0.706±0.303
*Grade	
WEL (47)	0.759±0.297
MOD (8)	0.787±0.316
POR (16)	0.511±0.262
*Subtype	
BAC (36)	0.766±0.302
ACI (12)	0.663±0.321
PAP (3)	0.762±0.253
SOL (12)	0.459±0.273
MUC (8)	0.851±0.212
SQC (38)	0.658±0.395
Grade	
WEL (0)	-
MOD (34)	0.649±0.415
POR (4)	0.736±0.145
LCC (18)	3.236±3.298
BRE (30) reference	1.007±0.130
Vascular involvement (127)	
Present (30)	0.872±0.916
Absent (97)	1.626±2.650
Lymphatic canal involvement (127)	
Present (25)	1.499±2.589
Absent (102)	0.941±1.135
Lymph node metastasis (114)	
Present (10)	0.706±0.341
Absent (104)	0.630±0.345
MIB1 labeling index (127)	
Low level (<10%) (36)	1.187±1.789
High level (≥10%) (91)	0.706±0.313
Oncogenic mutation (111)	
KRAS (5)	0.723±0.189
EGFR (22)	0.805±0.213
NONE (84)	0.662±0.382

*Significant in a one-way ANOVA analysis; BRE versus ADC, P<0.0001; BRE versus SQC, P<0.0001; BRE versus LCC, P = 0.0008; ADC/WEL versus ADC/POR, P = 0.0049; ADC/MOD versus ADC/POR, P = 0.0332; BAC versus SOL, P = 0.0013; PAP versus SOL, P = 0.0433; MUC versus SOL, P = 0.0007; ADC, adenocarcinoma; SQC, squamous cell carcinoma; LCC, large cell carcinoma; BRE, bronchial epithelia; WEL, well differentiated; MOD, moderately differentiated; POR, poorly differentiated carcinomas; BAC, bronchioloalveolar carcinoma; ACI, acinar adenocarcinoma; PAP, papillary adenocarcinoma; SOL, solid adenocarcinoma; MUC, mucinous adenocarcinoma; NONE, cases without KRAS or EGFR mutations.

## Discussion

An initial study on breast cancers suggested that miR-31 could be a tumor suppressor, which inhibited the invasive and metastatic spread of neoplastic cells [23]. Other studies have supported this initial finding and revealed the potential molecular mechanisms underlying how miR-31 evades invasion and metastasis [24]. MiR-31 was also shown to be downregulated in gastric cancers and was suggested to function as a tumor suppressor [25]. In contrast, miR-31 was found to be upregulated in colorectal cancers, and was suggested to promote the invasive and metastatic spread of neoplastic cells [26], [27]. Thus, the potential role of miR-31 in carcinogenesis may differ among the various types of cancers. In lung cancer, the expression of miR-31 has generally been reported to be higher in tumor tissue than in corresponding non-tumorous tissue [6]–[12]. Our results from quantitative RT-PCR analysis, in which several primary lung tumors were shown to strongly express miR-31, appeared to be consistent with the previous findings [6], [7], [9]–[12]. FISH analysis revealed that the amplification in the host gene locus could be one of the mechanisms responsible for the strong expression of miR-31. A recent study demonstrated a prognostic value of miR-31, as the higher expression of miR-31 was associated with a poorer outcome in lung cancers [13], and also suggested its oncogenic role through *in vitro* experiments [13]. Taken together with these findings, miR-31 was suggested to promote carcinogenesis, especially of large cell carcinomas. In contrast, miR-31 expression was markedly reduced or completely absent in some lung cancer cell lines. Moreover, its levels varied in primary lung tumors. Some tumors expressed miR-31 at lower levels than the corresponding non-tumorous tissue, while other tumors did not express it at all. Among the adenocarcinomas examined, the gene dosage was slightly lower in more aggressive tumors (poorly differentiated or solid subtype tumors). These results suggested that miR-31 may play a suppressive role in the carcinogenesis of adenocarcinomas. Thus, miR-31 may play a pleiotropic role in the development of lung cancers of different histological types. The expression of some known targets of miR-31, such as ITGA5, MMP16, and RHOA [28], was preliminarily examined in miR-31-transfected cells and lung cancer cells (Figure S3). However, the forced expression of miR-31 did not reduce the mRNA levels of these molecules. In native lung cancer cell lines, no correlation was observed between the level of these molecules and miR-31 levels among different histological types (Figure S3). Targets of miR-31 may vary in different situations, and complex crosstalk between the targets may lie hidden in lung cancer cells. Further studies that comprehensively investigate downstream targets are warranted in order to elucidate the potential molecular mechanism underlying how miR-31 promotes the carcinogenesis of different histological types of lung cancer.

Non-tumorous tissues exhibiting severe damage and inflammation strongly expressed miR-31. *In situ* hybridization analysis also confirmed the strong expression of miR-31 in regenerating non-neoplastic epithelial cells and mesenchymal cells. Thus, miR-31 may be induced by stimuli promoting cell growth in response to tissue damage and may control regenerative reactions. The altered expression of miR-31, whether it is downregulated or upregulation, may result in a disruption in the cellular homeostasis and may also participate in neoplastic transformation.

An analysis of the status of the gene locus demonstrated that the homozygous deletion of the miR-31 gene locus was one cause for the loss of its expression in lung cancer cell lines. However, some cell lines that retained the miR-31 gene locus also severely attenuated its expression. Previous studies reported that the hypermethylation of DNA or histone in the promoter locus of the miR-31 host gene was the cause for the severe downregulation of miR-31 in breast cancers [21] or adult T cell leukemia [29]. However, our results from the experiment using inhibitors for DNA methyltransferase and histone deacetylase did not support the involvement of epigenetic modification in the attenuation of miR-31 expression. Methylation-specific PCR and bisulfate sequencing analyses also failed to demonstrate a causative relationship between DNA hypermethylation of the promoter and such severe attenuation. A potential mechanism other than epigenetic modification is likely to be involved in the downregulation of miR-31 expression. The putative promoter locus of the miR-31 host gene contains multiple CCAAT enhancer elements [30]. CCAAT element binding protein (C/EBP)-β was found to induce miR-31 expression in airway epithelia in response to certain external stimuli [30]. C/EBP-α was shown to be severely downregulated in lung cancers [31]–[34], and may be the cause for the disordered expression of miR-31. An investigation on the possible involvement of the C/EBP family in the attenuation of miR-31 expression in lung cancer is warranted. Meanwhile, non-cancerous immortalized airway epithelial cell lines transformed by the SV40 large T antigen (NHBE-T, HPL1D, and HPL1A), in which p53 and RB-mediated pathways are inactivated, were found to express miR-31 at markedly lower levels than small airway epithelial cells (SAEC) and bronchial epithelial cells (NHBE). This viral antigen may directly and indirectly modulate the expression of miR-31.

In summary, the results of present study suggest that an altered expression of miR-31 due to either amplification or loss of its host gene locus may participate in lung carcinogenesis. To the best of our knowledge, this is the first study to describe the potential causative mechanism underlying the altered expression of miR-31 in lung cancers.

## Supporting Information

Figure S1 **The copy numbers of let-7i and U6 snRNA were measured using quantitative RT-PCR.** Let-7i levels were normalized to U6 snRNA levels. AEC, airway epithelial cells (non-cancerous cells); ADC, adenocarcinoma; SQC, squamous cell carcinoma; LCC, large cell carcinoma cell; SCC, small cell carcinoma.
(TIF)

Figure S2 **Biological effect of the restoration of miR-31 on a lung cancer cell line (LC2AD) is presented.** The pro-retrovirus vector pLHCX (BD Clontech) bearing DNA fragments including the miR-31 coding region (MIMAT0000089) flanking about a 100 base-pairs margin in both directions, was obtained. The retrovirus vectors (empty vector (MOCK), the sense strand of miR-31 (SS), and the antisense strand of miR-31 (AS)) were infected as the same method previously described [17]. Following a brief selection with Hygromycin B (BD Clontech), the surviving cells were harvested and counted, and 2.0×10^4^ were re-seeded onto a 10 cm dish. After 15 days, the cells were methanol-fixed and Giemsa-stained (A). The means and standard deviations (error bars) of colony counts from triplicate experiments are presented (B). Cells selected were grown and passed several times. Cumulated population doublings are presented (C). Cells harvested immediately after the selection process were examined for the expression of miR-31 and U6 snRNA by quantitative RT-PCR. The level of miR-31 normalized to that of U6 snRNA is presented (D).
(TIF)

Figure S3 **The copy numbers of ITGA5, RHOA, MMP-16, and GAPDH mRNA were measured using quantitative RT-PCR.** ITGA5 (A), RHOA (B), and MMP-16 (C) levels normalized to GAPDH levels are shown. AEC, airway epithelial cells (non-cancerous cells); TRAS, LC2AD lung cancer cell line - based transfectants (empty vector (MOCK), the sense strand of miR-31 (SS), and the antisense strand of miR-31 (AS)); ADC, adenocarcinoma; SQC, squamous cell carcinoma; LCC, large cell carcinoma cell; SCC, small cell carcinoma.
(TIF)
